# Supplementary material for: Effect of fetal malposition, primiparous, and premature rupture of membrane on Neonatal Near miss mediated by grade three meconium-stained amniotic fluids and duration of the active first stage of labor: Mediation analysis
Source: PLoS One. 2023 May 5;18(5):e0285280. doi: 10.1371/journal.pone.0285280 (PMC10162561; doi:10.1371/journal.pone.0285280)
Supplement: S1 Table — (DOCX) [file pone.0285280.s001.docx]

**Table 1**: The relationship between maternal related characteristics and Neonatal Near miss IN northwest Ethiopia, 2021(N=1277)

| Variables | Neonatal Near-miss | | COR(95% CI) | AOR(95% CI) |
| --- | --- | --- | --- | --- |
|  | Yes | No |  |  |
| Maternal education |  |  |  |  |
| Unable to read and write | 105 | 186 | 1.71(1.22-2.39),0.002 | 1.67(1.14-2.47)**0.009** |
| Read and Write | 47 | 150 | 0.95(0.64-1.43),0.804 | 0.85(0.62-1.25)0.352 |
| Primary school | 52 | 145 | 1.09(0.73-1.61),0.077 | 0.95(0.62-1.43)0.790 |
| Secondary school | 69 | 152 | 1.38(0.95-1.99),0.090 | 1.29(0.87-1.91)0.205 |
| College and above | 92 | 279 | **ref** | Ref. |
| Parity level |  |  |  |  |
| 1 | 203 | 365 | 1.84(1.28-2.63),0.001 | 2.48(1.63-3.79)**0.000** |
| 2-3 | 112 | 382 | 0.97(0.66-1.42),0.865 | 1.46(0.95-2.24)0.082 |
| 4 and above | 50 | 165 | **Ref.** | Ref. |
| Known HGB level at ANC |  |  |  |  |
| No | 305 | 680 | 1.73(1.26-2.38),0.001 |  |
| Yes | 60 | 323 | **Ref.** |  |
| Pregnancy induced HTN |  |  |  |  |
| Yes | 80 | 102 | 2.23(1.62-3.08) 0.000 | 2.10(1.49-2.95)**0.000** |
| No | 285 | 810 | **Ref.** | Ref. |
| Mode of admission |  |  |  |  |
| Self | 103 | 491 | **Ref.** | Ref. |
| Referral | 262 | 421 | 2.92(2.28-3.86) 0.000 | 2.28(1.88-3.28)**0.000** |
| PROM |  |  |  |  |
| Yes | 106 | 178 | 1.69(1.49-2.23) 0.000 | 1.47(1.09-1.98)**0.011** |
| No | 259 | 713 | **Ref.** | ref |
| Malposition |  |  |  |  |
| Yes | 32 | 40 | 2.10(1.29-3.39) 0.003 | 1.89(1.14-3.16)**0.014** |
