# Supplementary material for: Effect of fetal malposition, primiparous, and premature rupture of membrane on Neonatal Near miss mediated by grade three meconium-stained amniotic fluids and duration of the active first stage of labor: Mediation analysis
Source: PLoS One. 2023 May 5;18(5):e0285280. doi: 10.1371/journal.pone.0285280 (PMC10162561; doi:10.1371/journal.pone.0285280)
Supplement: S2 Table — (DOCX) [file pone.0285280.s002.docx]

**Table 2**: The relationship between obstetrics characterizes and duration of the active first stage of labor Northwest Ethiopia, 2021(n=1277).

| Variables | AFSOL | | COR(95%CI) | AOR(95%CI) |
| --- | --- | --- | --- | --- |
|  | <8hr | ≥8hrs |  |  |
| Maternal education |  |  |  |  |
| Unable to read and write | 189 | 102 | 1.16(0.85-1.60)0.356 |  |
| Read and Write | 105 | 92 | 0.72(0.51-1.02)0.061 |  |
| Primary school | 126 | 71 | 1.11(0.78-1.59)0.558 |  |
| Secondary school | 130 | 91 | 0.89(0.64-1.26)0.526 |  |
| College and above | 228 | 143 | ref. |  |
| Parity level |  |  |  |  |
| 1 | 312 | 256 | 0.52(0.37-0.72)**0.000** | 0.51(0.36-0.71)**0.000** |
| 2-3 | 315 | 179 | 0.75(0.53-1.05)0.096 | 0.75(0.52-1.06)0.100 |
| 4 and above | 151 | 64 | ref. |  |
| Known HGB level at ANC |  |  |  |  |
| No | 607 | 378 | 1.14(0.87-1.48)0.346 |  |
| Yes | 171 | 121 | ref. |  |
| Mode of admission |  |  |  |  |
| Self |  |  | ref. |  |
| Referral |  |  | 0.82(0.65-1.03)0.082 |  |
| Pregnancy induced HTN |  |  |  |  |
| Yes | 672 | 423 | 1.13(0.83-1.56)0.423 |  |
| No | 106 | 76 | ref. |  |
| PROM |  |  |  |  |
| No | 587 | 406 | ref. | ref. |
| Yes | 191 | 93 | 1.42(1.08-1.88)**0.013** | 1.49(1.13-1.98)**0.005** |
| Malposition |  |  |  |  |
| No | 743 | 462 | 1.70(1.06-2.74)**0.029** | 1.82(1.11-2.94)**0.017** |
| Yes | 35 | 37 | ref. |  |
