# Supplementary material for: Effect of fetal malposition, primiparous, and premature rupture of membrane on Neonatal Near miss mediated by grade three meconium-stained amniotic fluids and duration of the active first stage of labor: Mediation analysis
Source: PLoS One. 2023 May 5;18(5):e0285280. doi: 10.1371/journal.pone.0285280 (PMC10162561; doi:10.1371/journal.pone.0285280)
Supplement: S3 Table — (DOCX) [file pone.0285280.s003.docx]

**Table 3:** Relationship between obstetrics characterizes and grade III meconium stained amniotic fluid Northwest Ethiopia, 2021(n=1277).

| Variables | GIIIMSAF | | COR(95%CI) | AOR(95%CI) |
| --- | --- | --- | --- | --- |
|  | Yes | No |  |  |
| Maternal education |  |  |  |  |
| Unable to read and write | 26 | 265 | 1.04(0.61-1.78)0.889 |  |
| Read and Write | 15 | 182 | 0.87(0.46-1.66)0.677 |  |
| Primary school | 25 | 172 | 1.54(0.88-2.68)0.127 |  |
| Secondary school | 18 | 203 | 0.94(0.51-1.72)0.839 |  |
| College and above | 32 | 339 | ref. |  |
| Parity level |  |  |  |  |
| 1 | 82 | 485 | 3.45(1.76-6.80)0.000 | 3.47(1.75-6.87)**0.000** |
| 2-3 | 24 | 470 | 1.05(0.49-2.23)0.906 | 1.23(0.53-2.65)0.592 |
| 4 and above | 10 | 205 | ref. | **ref.** |
| Known HGB level at ANC |  |  |  |  |
| No | 12 | 100 | 2.75(1.49-5.08)**0.001** | 1.92(1.02-3.62)**0.045** |
| Yes | 104 | 1061 | ref. | ref. |
| Pregnancy induced HTN |  |  |  |  |
| No | 104 | 991 | ref. |  |
| Yes | 12 | 170 | 0.67(0.36-1.25)0.209 |  |
| Mode of admission |  |  |  |  |
| Self | 27 | 567 | ref. | ref. |
| Referral | 89 | 594 | 3.15(2.01-4.91)0.000 | 2.44(1.53-3.88)**0.000** |
| PROM |  |  |  |  |
| No | 86 | 907 | Ref. |  |
| Yes | 30 | 254 | 1.25(0.80-1.93)0.326 |  |
| Malposition |  |  |  |  |
| No | 103 | 1102 | ref. | ref. |
| Yes | 13 | 59 | 2.35(1.25-4.44)**0.008** | 2.12(1.09-4.10)**0.029** |
