# Supplementary material for: Effect of fetal malposition, primiparous, and premature rupture of membrane on Neonatal Near miss mediated by grade three meconium-stained amniotic fluids and duration of the active first stage of labor: Mediation analysis
Source: PLoS One. 2023 May 5;18(5):e0285280. doi: 10.1371/journal.pone.0285280 (PMC10162561; doi:10.1371/journal.pone.0285280)
Supplement: Attachment — Submitted filename: Response to Reviewers.docx [file pone.0285280.s005.docx]

Felix Chikaike Clement Wekere

Academic Editor
PLOS ONE

November, 2022

**Subject: Rebuttal letter to editor and reviewers**

Dear Dr. Felix Chikaike Clement Wekere

Thank you for inviting us to submit a revised draft of our manuscript (PONE-D-22-20623) entitled *“Effect of fetal malposition, primiparous, and premature rupture of membrane on Neonatal Near miss mediated by grade three meconium-stained amniotic fluids and duration of the active first stage of labour: Mediation analysis*” to Journal of PLOS ONE. This research is funded by University of Gondar with reference number of (6-9/03/2013). The founder had no role in the design of the study, data collection, analysis, in writing of the manuscript and decision to publish. Files containing supporting information have been submitted.We appreciate the time and effort you and the reviewers have dedicated to providing insightful feedback on ways to strengthen our paper. Thus, it is with great pleasure that we resubmit our manuscript for further consideration. We have incorporated changes that reflect the detailed suggestions you have graciously provided. We also hope that our edits and the responses we provide below satisfactorily address all the issues and concern you and the reviewers have noted. To facilitate your review of our revisions, the following is a point-by-point response to the questions and comments delivered in your letter dated November 22, 2022.

Sincerely,

Mengstu Melkamu Asaye

| S-no | **Editor suggestions** | **Authors’ response** |
| --- | --- | --- |
| 1 | Additionally, ensure you proofread and edit the manuscript for typographical and grammatical errors throughout the manuscript. | We sincerely appreciate your efforts and encouragements to produce a high-quality scientific paper for the scientific community. We have a high regard for your journal (PLOS ONE) because of the emphasis you placed on quality and scientifically sound articles.  Dear Editor, I appreciate your detailed comments and prompt response. The manuscript has been reviewed for grammatical and punctuation errors in each line and page, and necessary changes have been made in the main document. |
| 2 | Line 36, write the correct meaning of the abbreviation AOR. Kindly note AOR means (adjusted odds ratio). | Thank you once more. Based on your suggestion, it has been corrected in the main document. |
| 3 | Line 38- ‘The proportion of neonatal near-misses was 28.6%’, In addition to the percentage add the count or number for instance 26% (X/Y). In the abstract, state the statistical software you used in analysing the data as well as the version, city, and country of the manufacturer. | It's an important point you brought up. It has been corrected in the main document. |
| 3 | Lines 41&42 -Correct punctuations,Line 98- the sentence is incomplete kindly revise | Thank you once more. The main document has been updated to correct this. |
| 4 | Line 122 - ‘The sample size was determined using a single population proportion formula.’ state the formula for reproducibility | It is corrected as “The sample size was determined using a single population proportion formula for reproducibility with the following statistical assumptions:…” based on your valuable comment. |
| 5 | Revise lines 127 to 130 by stating your sampling method first and then how it was carried out -for clarity. Line 128 ‘The number of skips was four’ do you mean the sampling interval? | Thank you once more, dear editor. You raised an important point that will help to improve the paper even more. It has been corrected as “Six public health hospitals were selected randomly using a lottery method. The calculated sample size was proportionally allocated to each hospital based on their previous six-month reports. The number of sampling interval was four. Using a lottery method, the first case in each hospital was selected at random. Finally, using a systematic random sampling technique, live-born newborns were selected” in the main document. Yes it was to mean sampling interval and edited in the document. |
| 6 | Line 142 -use ‘research assistants’ rather than ‘data collectors’ | Based on your suggestion, it has been changed in the main document to "research assistance." |
| 7 | In Line 190 kindly revise the sentence. Also, edit lines 203 and 249 for grammar and punctuation amongst others. | Thank you once more for your thorough review and insightful comments. The grammatical and punctuation errors have been corrected and incorporated into the main document as a result of your comments. |
| s-no | **Reviewers’ suggestion** | **Authors’ response** |
| 1 | Reviewer #1: ABSTRACT : The information in the abstract was well written. The background and methods section were well captured. However under the results section, only the overall proportion of neonatal near- misses was captured included the AOR of the various determinants. It will be worth mentioning was percentages were attributed to the various obstetric determinants. | Dear reviewer, first and foremost, please accepts my heartfelt gratitude for your insightful feedback and comments. You brought up an extremely important point. We wanted to see the overall association through adjusted odds ratio rather than percentage of each attribute in the cross-sectional study. |
| 2 | Background ‘The proportion was 22% in northwestern Brazil [4], 17.2% in Australia [5], 86% in central 4 65 Gujarat, India [6], and 79% in Nepal [7].In three Africa countries like Benin, Burkina Faso, and 66 Morocco, it was 27.1%, 19.1%, and 30.4%, respectively [3], and 36.7% in central Uganda [8]’………..Are the various proportions mentioned nationwide values or in selected facilities? Its unclear…..Kindly clarify. | Dear reviewer, You are entirely correct. Thank you once more. It was facility-level studies. The main document has been updated to read "in facility-based studies." |
| 3 | Paragraph 4: ‘The entry of meconium into the amniotic fluid inside the uterus is thought to cause fetal distress by causing fetal hypoxia’…….fetal distress from fetal hypoxia causes fetus to pass meconium and not vice versa… meconium passage doesn’t cause fetal hypoxia… Kindly correct that | Dear reviewer, first and foremost, gratitude You are entirely corrects. Thank you once more. Thank you so much for bringing this to our attention.  In the main document, it has been corrected as "The entry of meconium into the amniotic fluid inside the uterus is thought to cause fetal distress." |
| 4 | Operational definitions: Kindly review the grammar …’ Neonatal Near Miss (NNM) is defined as when newborns with severe complications who 182 nearly died but survived at birth and who faced to at least one of the Neonatal Near Miss 183 Assessment scale (NNMAS) elements is referred to as a Neonatal Near Miss ‘ | Dear reviewer, based on your insightful comments, it has been updated in the main document to read as “Neonatal Near Miss (NNM) is defined as neonates with severe complications who nearly died but survived immediately after birth in selected hospitals labour wards or on the first day of admission to a neonatal intensive care unit after being born in such hospitals, and who met at least one of the validated Neonatal Near-Miss Assessment Scale criteria” |
| 5 | Discussion Paragraph 3: ‘This finding was also lower when compared to studies conducted in Brazil [44], central 358 Gujarat- India [6],Nepal ……’ Which finding are you making reference to? Kindly clarify and ensure Kindly review your grammar throughout the work as some sentences in your discussion begin with ‘ because’ | Thank you once more. It was supposed to mean (28.6%). Based on your feedback, it has been corrected in the main document as "This finding, on the other hand, was lower when compared to studies conducted in Brazil..."  Each line and page of the manuscript has been double-checked for grammatical and punctuation errors, and significant changes have been made in response to your insightful feedback. |

**Thank you so much**
