# Supplementary material for: Effect of fetal malposition, primiparous, and premature rupture of membrane on Neonatal Near miss mediated by grade three meconium-stained amniotic fluids and duration of the active first stage of labor: Mediation analysis
Source: PLoS One. 2023 May 5;18(5):e0285280. doi: 10.1371/journal.pone.0285280 (PMC10162561; doi:10.1371/journal.pone.0285280)
Supplement: Attachment — Submitted filename: PLOS REVIEW ON MEDIATION ANALYSIS OF NEONATAL NEAR MISS.docx [file pone.0285280.s006.docx]

**The Editorial Manager PLOS ONE,**

Thank you for asking me to review the manuscript titled ***“EFFECT OF FETAL MALPOSITION, PRIMIPAROUS, AND PREMATURE RUPTURE OF MEMBRANES ON NEONATAL NEAR MISS MEDIATED BY GRADE THREE MECONIUM-STAINED AMNIOTIC FLUIDS AND DURATION OF ACTIVE FIRST STAGE OF LABOR: MEDIATION ANALYSIS”.***

In reviewing the manuscript, i noted the comments of the previous REVIEWERS and the responses of the AUTHOURS to the issues raised by the REVIEWERS.

However, i observed that there were still some typographical, spelling, and grammatical errors which the previous Reviewers had expected the Authors to address but which were not addressed. Having painstakingly gone through the manuscript, I have tried to highlight some of the perceived errors for ease of reference and correction by the Authors.

On the whole, the study is a good one as it tried to highlight some of the major contributors to Neonatal Near Misses some of which contribute significantly to both maternal and perinatal mortality if not managed properly, especially in low-income resource areas.

1. **Abstract: Background:**
   a. Page 2: Lines 29: …… limited *in* Ethiopia
2. **Abstract: Methods:**
   a. Page 2: line 34: ……and a review of medical records *were* used to….
   b. Page 2: line 36: …. via mediators *were* examined
   c. Page 2: line 37: ....and β-coefficients *were* calculated…
   d. Page 3: lines 56-57: to read *“Early diagnosis of these potential danger signs and appropriate intervention could be of supreme importance in reducing NNM”.*
   e. Page 4: line 69: delete *“was”* after Ethiopia.
   f. Page 5: line 97: delete *“on”* after followed up.
   g. Page 5: line 107: delete *“at”* after investigate.
3. **Methods and Materials: Study design, setting and period:**
   a. Page 6: Line 116: to read: …the newborn ward *“is”* also divided…
   b. Page 6: Line 117: midwife to read *“Midwives”.*
   c. Page 6: Line 118-119: The sentence *“Of these, 15,000 deliveries at maternity wards”* does not make sense and should be reviewed! *Are the Authors saying that there were 15,000 deliveries in all the maternity wards used for the study?*
   d. Page 6: Line 120**: delete *~~“born to women~~”*! to read *“The medical records of five newborns were incomplete”*.**
4. **Variables and data collection procedures:**
   a. Page 7: Line 145: To read ***“three supervisors “were”***
   b. Page 7: Line 146-147: To read: ***“The data collection process was monitored daily and the acquired data were reviewed for accuracy and consistency. One computer was* *used for data entry.***
   c. Page 8: Line 165***: ….and entered “into” Epi-Info 7.1.2.***
   d. Page 8: Line 172: ***inflation*** *factor was* less than 10.
5. **Ethical Approval:**
   a. Page 9: Line 1179: ***“Support letter”*** to read ***“Support letters”***
   b. Page 9: Line 183: ***“as well*** as***….***
   c. Page 9: Line 190: PIH *was defined as a new onset* of hypertension…
6. **Results:**
   a. Page 10: Line 209: **“couldn’t”** to read: “could not”
   b. Page 10: Line 210-212: to read: ***Mothers of babies who had a college education or above constituted 371 (29%) of cases, 302 (24%) were government employed, while 449 (35.2%) had a monthly income that fell within the poor group* (Table 1).**
   c. **Table 1: Page 12:** Average family monthly income was classified as: **Poor, Medium and Reach**. It is not clear what the word “*Reach”* means or implies!
   d. **Page 226 under Table 1 NB: ****Muslim and protestant and**…(Please review)**
7. **Obstetrics related characteristics:**
   a. Page 12: Line 228-240: to read: ***In this study, 1186 (93%) of the women received antenatal care, with 258 (21.8%) having fewer than four antenatal contacts. A total of 1107 (86.7%) of the mothers received iron and foliate supplements during pregnancy. The results also revealed that 568 (44.5%) of the mothers were primiparous, while 1106 (86.6%) had a planned pregnancy. Short birth intervals were observed in 160 (22.6%) of the mothers, while 37 (2.9%) had stillbirths and 13 (1%) had neonatal deaths. The hemoglobin levels during pregnancy were unknown in 112 (8.8%) of the mothers. Among the study participants, 284 women representing 22.2% had premature rupture of the membranes (PROM), 14.3% experienced pregnancy induced hypertension (PIH) while 683 (53.5%) were referred to the hospitals from other health facilities. The results also showed that 499 (39.1%) of the study participants completed their active first stage of labour within 8 hours or less, 78 (6.1%) encountered fetal malposition during labour and birth, 91% had grade three meconium-stained amniotic fluids, while 568 (44.5%) gave birth via caesarean section*** (Table 2).
8. **Neonatal near-miss and its determinants:**
   a. Page 14: line 256: **“couldn’t”** to read “could not”
   b. Page 14: line 258: **“associated to”** to read “associated with”
   c. Page 14: line 259: …….fetal malposition “*were”* significantly…
   d. Page 14: line 260: **“associated to the ~~mediator~~ duration of the AFSOL”…** to read *“associated with” the duration of the AFSOL….*
   e. Page 14: line 262: delete ***~~“mediator~~” before grade 3….***
   f. Page 14: line 268: to read …………as compared to *those who did not have PIH.*
   g. Page 14: line 273: **“a preterm rupture”** to read *“a premature rupture”*
   h. Page 15: line 284-285: review to read *“the study showed that the duration of AFSOL and G111MSAF were the obstetric factors that mediated NNM”.*
   i. Page 15: line 291: delete “**~~neonates~~’” and replace with *“the”* primiparous mothers….**
   j. Page 15: line 292: replace **“early** rupture” with *“premature* rupture”
   k. Page 15/16: line 295-297: delete ~~“when compared to”~~ to read *“Mothers of newborns who did not have fetal malposition during labour and birth were 44% less likely to have AFSOL in less than 8 hours than those who had fetal malposition (AOR = 0.56, 95% CI: 0.34-0.91).* (Table 3)
   l. Page 16: line 312: study *also* revealed that *being* primiparous….
   m. Page 16: line 313: …. “**Primiparous had also a positive**”… to read “*Primiparity also had a positive*”…
9. **Discussion:**
   a. Page 19: line 356: The study revealed *that…..*
   b. Page 19 line 357: to read: “studies conducted in *other health facilities* in Northeastern Brazil…..
   c. Page 19: line 358: to read:  *“This ~~cause~~* could be *due to* a disparity in the quality of care provided in *the health facilities in these countries”. Delete ~~to individuals on the verge of death but survived newborns~~*
   d. Page 19: line 359-361: to read ***“In addition, prospective follow-up was used in the Brazil and Australia studies, and the outcome was determined using a different measurement scale, whereas in our study, we used a contextualized [38}, valid, and reliable NNMAS [39].***
   e. Page 19: line 362: …….”was consistent with *other* *studies from Brazil [46] and the Jima Zone, Southwest Ethiopia [12]*”
   f. Page 19: line: 364: to read: *“The NNM rate of 28.6% in our study was, however, lower when compared to other studies conducted in health facilities in Brazil [44], central Gujarat India [6], Nepal [7] and Central Uganda [45}, Hawasa Ethiopia [46], south Ethiopia [14], and Debre Tabor hospital, Northwest Ethiopia [13]. ~~Study participants~~ Participants in these other studies in Brazil, central Gujarat India, and Uganda, were neonates born to women hospitalized with severe obstetric problems.*
   g. Page 20: line: 369: Add “were” after Debre Tabor.
   h. Page 20: line: 375: to read: “In contrast, in the studies conducted in Nepal……..
   i. Page 20: line: 380: In ~~this finding~~ our study, newborns born to women who ~~couldn’t~~ *could not…..*
   j. Page 20: line 382: ~~According to this study,~~ *The findings in our study show that the chances …….*
   k. Page 20: line 387-391: to read: *This is consistent with the findings in* a study conducted at Debre Tabor Hospital in Northwest Ethiopia [13], *and tends to suggest* that psychological concerns, stress, and intense labour pains *may* have a significant impact on primiparous women’s energy and power during labour and birth. This *may result in prolonged* labour which has negative consequences for the newborn [20]. So, while being multiparous *is risky to the* mother, the infant being born *to a* primiparous *mother* is even *at a greater risk*.
   l. Page 21: line 395: to ***~~their counterparts~~*** read: ….This finding is similar to the findings reported in studies conducted in Brazil [17],……….
   m. Page 21: line 396-397: to read: ……. *This could be* ***~~because~~*** *attributed to intrauterine growth retardation (IUGR), placental insufficiency, and increased interventional delivery that may be performed regardless of the gestational age.*
   n. Page 21: line 402: to read: ……… *than those who were not referred.*
   o. Page 20: line 406: to rea: ***~~Based on this~~*** *It is, therefore, recommended that midwives should prioritize providing timely treatment to those vulnerable newborns.*
   p. Page 21: line 408: to read: The study ***~~suggested~~*** revealed that…….
   q. Page 21: line 409: to read: ….. than ***~~their peers~~*** those mothers who did not have PROM….
   r. Page 21: line 410: replace “accordance” with “agreement”
   s. Age 22: line 416 -417: to read:…….than ***~~their counterparts~~*** those without fetal malposition. ***~~This is a warning to~~*** This finding should serve as a guide to midwives to spot ***~~it early and consult an obstetrician to take~~*** the danger early and call for immediate action to save the lives of the newborns.
   t. Page 22: line 419: *“1.97-fold increased”* to read *“1.97-fold increase” in the risk of NNM.*
   u. Line 420: delete *“****~~than their peers”~~***
   v. **~~This finding was supported by”~~** to read: *This finding is consistent with a study in India which found that highly thick meconium-stained amniotic fluid is a sign of fetal distress [29]*. Delete ***~~“which is concerning to the neonate’s better prognosis’~~***
   w. Page 22: line: 426: to read: …… ***This is consistent with the findings in our study which showed that neonates born to women who had AFSOL lasting more than 8 hours had a 1.81-fold increased risk of NNM than those born to women who had AFSOL lasting less than 8 hours.*** Delete ~~than their counterparts~~
   x. Page 22: line 428: to read: “*This* ***is”*** ***to read*** *“This may be”…..*
   y. Page 22: line 429: to read: …….. *“The most important strategy for* ***~~intervening~~*** *early detection of prolonged AFSOL is the consistent use and interpretation of the partograph.*
   z. Page 23: line 439: replace *“endorses”* with *“supported”*. Delete: ~~“This is the responsibility of the midwives and obstetricians who work.~~ **It does not make any sense here!.**
   aa. Page 23 line 442: *“lasting”* to read *“going through”*
   bb. Page 23: line 448: *“indirectly”* to read *“indirect”*
   cc. Page 23: line 450: **delete the whole sentence**
   dd. **Page 23: line 454: ~~Ethiopian studies supports~~*This finding is supported by other studies conducted in Ethiopia [13, 19]***
   ee. **Page 24:** line 462: “this idea” to read “this finding”
   ff. **Page 24: line 466-467: to read:** “*The strength of this study lies in the fact that it is a multi-centre study with a large sample size. In addition, a psychometrically validated scale was used to assess the outcome.* **The study also determined the direct ………..**
   gg. **Page 24: line 471-473: …….***were ~~“missed~~” excluded and only live births ~~babies~~ were studied in the early neonatal period with no follow-up. We strongly recommend that a study be conducted to cover the entire neonatal period.*
   hh. **Page 25:** *line 478: replace: “nearly dead but living newborn” with “moribund newborn”.*
   ii. **Page 25;** *line 479: to read: “educate midwives, doctors and other health professionals on how to manage limited resources* ***~~in order~~*** *to improve quality of care and reduce NNM and mortality.*
10. **Conclusion:**
    a. **Page 25 line482: “couldn’t” to read “could not”**
    b. **Page 25: line 488: ….. “could be of supreme importance in reducing NNM.”**
