# Supplementary material for: Effect of fetal malposition, primiparous, and premature rupture of membrane on Neonatal Near miss mediated by grade three meconium-stained amniotic fluids and duration of the active first stage of labor: Mediation analysis
Source: PLoS One. 2023 May 5;18(5):e0285280. doi: 10.1371/journal.pone.0285280 (PMC10162561; doi:10.1371/journal.pone.0285280)
Supplement: Attachment — Submitted filename: Response to Reviewers.docx [file pone.0285280.s007.docx]

Felix Chikaike Clement Wekere

Academic Editor
PLOS ONE

March, 2023

**Subject: Rebuttal letter to editor and reviewers**

Dear Dr. Felix Chikaike Clement Wekere

Thank you for inviting us to submit a revised draft of our manuscript (PONE-D-22-20623) entitled *“Effect of fetal malposition, primiparous, and premature rupture of membrane on Neonatal Near miss mediated by grade three meconium-stained amniotic fluids and duration of the active first stage of labour: Mediation analysis*” to Journal of PLOS ONE. This research is funded by University of Gondar with reference number of (6-9/03/2013). The founder had no role in the design of the study, data collection, analysis, in writing of the manuscript and decision to publish. Files containing supporting information have been submitted. We appreciate the time and effort you and the reviewers have dedicated to providing insightful feedback on ways to strengthen our paper. Thus, it is with great pleasure that we resubmit our manuscript for further consideration. We have incorporated changes that reflect the detailed suggestions you have graciously provided. We also hope that our edits and the responses we provide below satisfactorily address all the issues and concern you and the reviewers have noted. To facilitate your review of our revisions, the following is a point-by-point response to the questions and comments delivered in your letter dated March 30, 2023 22.

Sincerely,

Mengstu Melkamu Asaye

| S-no | **Editor suggestions** | **Authors’ response** |
| --- | --- | --- |
| 1 | The reviewers have raised important concerns about some aspects of the methodology and presentation, which you should address carefully in your revised manuscript. Kindly address each point carefully in your response to the reviewers’ document and revise the manuscript accordingly.  Additionally, ensure you proofread and edit the manuscript for syntax and grammatical errors throughout the manuscript. Please, read through the review comments and suggestions made painstakingly and make the corrections to improve your manuscript. | We sincerely appreciate your efforts and encouragements to produce a high-quality scientific paper for the scientific community. We have a high regard for your journal (PLOS ONE) because of the emphasis you placed on quality and scientifically sound articles.  Dear Editor, I appreciate your facilitation and prompt response. The manuscript has been reviewed for grammatical and punctuation errors in each line and page, and necessary changes have been made in the main document. |
| s-no | **Reviewers’ suggestion** | **Authors’ response** |
|  |  | **Dear reviewer**  Thank you so much for your insightful comments, grammatical and topographic corrections. |
| 1 | Abstract: Background:   a. Page 2: Lines 29: …… limited in Ethiopia | Thank you so much. It has been corrected as “However, studies causal pathway determinants are limited in Ethiopia” in the main document. |
| 2 | **Abstract: Methods:**   a) Page 2: line 34: ……and a review of medical records were used to…. b) Page 2: line 36: …. via mediators were examined c) Page 2: line 37: ....and β-coefficients were calculated… d) Page 3: lines 56-57: to read “Early diagnosis of these potential danger signs and appropriate intervention could be of supreme importance in reducing NNM”. e) Page 4: line 69: delete “was” after Ethiopia. f) Page 5: line 97: delete “on” after followed up. g) Page 5: line 107: delete “at” after investigate. | Thank you again. It is corrected in each line(34,36 and 37) of the main document.  It has been incorporated as” Early diagnosis of these potential danger signs and appropriate intervention could be of supreme importance in reducing NNM” in the main document at line 52-56 line.  It is corrected in each highlighted page and line of the main document. |
| 3 | Methods and Materials: Study design, setting and period:   a) Page 6: Line 116: to read: …the newborn ward “is” also divided… b) Page 6: Line 117: midwife to read “Midwives”. c) Page 6: Line 118-119: The sentence “Of these, 15,000 deliveries at maternity wards” does not make sense and should be reviewed! Are the Authors saying that there were 15,000 deliveries in all the maternity wards used for the study? d) Page 6: Line 120: delete “born to women”! to read “The medical records of five newborns were incomplete”. | Dear reviewer, thank you very much for your insightful comments. It is corrected as “The newborn ward is also divided into sections. Each ward had senior doctors, residents, midwives, and nurses” in the main document at line 115-116.  c. We appreciated your insightful remarks. The magnitude also corrected and incorporated  in the main document as” Annually, of 13,640 deliveries at maternity wards, 2340 neonates were admitted to neonatal units”from line 117-118.  d. Data on live born newborns and medical records were collected. The medical records of five newborns were incomplete. |
| 4 | **Variables and data collection procedures:**   a) Page 7: Line 145: To read **“three supervisors “were”** b) Page 7: Line 146-147: To read: **“The data collection process was monitored daily and the acquired data were reviewed for accuracy and consistency. One computer was used for data entry.** c) Page 8: Line 165**: ….and entered “into” Epi-Info 7.1.2.** d) Page 8: Line 172: **inflation** factor was less than 10. | Dear, reviewer  Thank you for your helpful and constructive grammatical corrections. It has been corrected as “ Nine research assistants and three supervisors were involved in the data collection line 145”,  “The data collection process was monitored daily and the acquired data were reviewed for accuracy and consistency. One computer was used for data entry.” In the main document line 146-147.  “Data were manually checked, coded, and entered into Epi-Info 7.1.2” incorporated in the main document line 164. |
| 5 | **Ethical Approval:**   a) Page 9: Line 1179: “Support letter” to read “Support letters” b) Page 9: Line 183: “as well as…. c) Page 9: Line 190: PIH was defined as a new onset of hypertension… | Dear reviewer  Thank you again for the correction. It has been incorporated as “Support letters “line 178,  “The questionnaires, as well as all data collected from newborn mothers and reviews, were kept in a secure location and used solely for research purposes” in the main document.  “Pregnancy-Induced Hypertension (PIH) was defined as a new onset of hypertension” line 189 in the main document. |
| 6 | **Results:**   a) Page 10: Line 209: “couldn’t” to read: “could not” b) Page 10: Line 210-212: to read: Mothers of babies who had a college education or above constituted 371 (29%) of cases, 302 (24%) were government employed, while 449 (35.2%) had a monthly income that fell within the poor group (Table 1). c) Table 1: Page 12: Average family monthly income was classified as: Poor, Medium and Reach. It is not clear what the word “Reach” means or implies! d) Page 226 under Table 1 NB: **Muslim and protestant and…(Please review) | Thank you again. It is corrected in the main document as “could not”  “Mothers of babies who had a college education or above constituted 371 (29%) of cases, 302 (24%) were government employed, while 449 (35.2%) had a monthly income that fell within the poor group (Table 1” line 210-12 on the main document.  “Reach” it was mistakenly reported due to type error. It has been changed to “rich” in the main document table 1  “and” deleted in the main document. |
| 7 | **Obstetrics related characteristics:**   a) Page 12: Line 228-240: to read: In this study, 1186 (93%) of the women received antenatal care, with 258 (21.8%) having fewer than four antenatal contacts. A total of 1107 (86.7%) of the mothers received iron and foliate supplements during pregnancy. The results also revealed that 568 (44.5%) of the mothers were primiparous, while 1106 (86.6%) had a planned pregnancy. Short birth intervals were observed in 160 (22.6%) of the mothers, while 37 (2.9%) had stillbirths and 13 (1%) had neonatal deaths. The hemoglobin levels during pregnancy were unknown in 112 (8.8%) of the mothers. Among the study participants, 284 women representing 22.2% had premature rupture of the membranes (PROM), 14.3% experienced pregnancy induced hypertension (PIH) while 683 (53.5%) were referred to the hospitals from other health facilities. The results also showed that 499 (39.1%) of the study participants completed their active first stage of labour within 8 hours or less, 78 (6.1%) encountered fetal malposition during labour and birth, 91% had grade three meconium-stained amniotic fluids, while 568 (44.5%) gave birth via caesarean section (Table 2). | a) Oh, I'm at a loss for words to express how comprehensive and informative your edition is. It has been incorporated as “In this study, 1186 (93%) of the women received antenatal care, with 258 (21.8%) having fewer than four antenatal contacts. A total of 1107 (86.7%) of the mothers received iron and foliate supplements during pregnancy. The results also revealed that 568 (44.5%) of the mothers were primiparous, while 1106 (86.6%) had a planned pregnancy. Short birth intervals were observed in 160 (22.6%) of the mothers, while 37 (2.9%) had stillbirths and 13 (1%) had neonatal deaths. The hemoglobin levels during pregnancy were unknown in 112 (8.8%) of the mothers. Among the study participants, 284 women representing 22.2% had premature rupture of the membranes (PROM), 14.3% experienced pregnancy induced hypertension (PIH) while 683 (53.5%) were referred to the hospitals from other health facilities. The results also showed that 499 (39.1%) of the study participants completed their active first stage of labour within 8 hours or less, 78 (6.1%) encountered fetal malposition during labour and birth, 91% had grade three meconium-stained amniotic fluids, while 568 (44.5%) gave birth via caesarean section (Table 2)” line 226 -237 in the main document. |
| 8 | **Neonatal near-miss and its determinants:**   a) **Page 14: line 256: “couldn’t” to read “could not”** b) Page 14: line 258: “associated to” to read “associated with” c) Page 14: line 259: …….fetal malposition “were” significantly… d) Page 14: line 260: “associated to the mediator duration of the AFSOL”… to read “associated with” the duration of the AFSOL…. e) Page 14: line 262: delete “mediator” before grade 3…. f) Page 14: line 268: to read …………as compared to those who did not have PIH. g) Page 14: line 273: “a preterm rupture” to read “a premature rupture” h) Page 15: line 284-285: review to read “the study showed that the duration of AFSOL and G111MSAF were the obstetric factors that mediated NNM”. i) Page 15: line 291: delete “neonates’” and replace with “the” primiparous mothers…. j) Page 15: line 292: replace “early rupture” with “premature rupture” k) Page 15/16: line 295-297: delete “when compared to” to read “Mothers of newborns who did not have fetal malposition during labour and birth were 44% less likely to have AFSOL in less than 8 hours than those who had fetal malposition (AOR = 0.56, 95% CI: 0.34-0.91). (Table 3) l) Page 16: line 312: study also revealed that being primiparous…. m) Page 16: line 313: …. “Primiparous had also a positive”… to read “Primiparity also had a positive”… | Dear reviewer  Thank you for your time and helpful grammatical and contextual corrections. All (a,b,c,d,e,f, and g) comments has been incorporated in the main document.  “The study showed that the duration of AFSOL and G111MSAF were the obstetric factors that mediated NNM” in the main document line 283-284.  It has been corrected as “primiparous mothers...”291 in the main document  It has been corrected in the main documents “Mothers of new-borns who did not have fetal malposition during labour and birth were 44% less likely to have AFSOL in less than 8 hours than those who had fetal malposition (AOR = 0.56, 95% CI: 0.34-0.91). (Table 3)  It has been corrected as “Primiparity also had a positive and significant direct relationship with GIIIMSAF (ß=1.241, P<0.001) and NNM (ß=1.938, P<0.001) ‘ line 309-310 in the main document. |
| 9 | **Discussion:** | Dear reviewer thank you again for your valuable edition and corrections |
|  | a) **Page 19: line 356: The study revealed that…..** b) Page 19 line 357: to read: “studies conducted in other health facilities in Northeastern Brazil….. c) Page 19: line 358: to read: “This ~~cause~~ could be due to a disparity in the quality of care provided in the health facilities in these countries”. Delete ~~to individuals on the verge of death but survived newborns~~ d) Page 19: line 359-361: to read **“In addition, prospective follow-up was used in the Brazil and Australia studies, and the outcome was determined using a different measurement scale, whereas in our study, we used a contextualized [38}, valid, and reliable NNMAS [39].** e) Page 19: line 362: …….”was consistent with other studies from Brazil [46] and the Jima Zone, Southwest Ethiopia [12]” f) Page 19: line: 364: to read: “The NNM rate of 28.6% in our study was, however, lower when compared to other studies conducted in health facilities in Brazil [44], central Gujarat India [6], Nepal [7] and Central Uganda [45}, Hawasa Ethiopia [46], south Ethiopia [14], and Debre Tabor hospital, Northwest Ethiopia [13]. ~~Study participants~~ Participants in these other studies in Brazil, central Gujarat India, and Uganda, were neonates born to women hospitalized with severe obstetric problems. g) Page 20: line: 369: Add “were” after Debre Tabor. h) Page 20: line: 375: to read: “In contrast, in the studies conducted in Nepal…….. i) Page 20: line: 380: In ~~this finding~~ our study, newborns born to women who ~~couldn’t~~ could not…. j) Page 20: line 382: ~~According to this study,~~ The findings in our study show that the chances ……. k) Page 20: line 387-391: to read: This is consistent with the findings in a study conducted at Debre Tabor Hospital in Northwest Ethiopia [13], and tends to suggest that psychological concerns, stress, and intense labour pains may have a significant impact on primiparous women’s energy and power during labour and birth. This may result in prolonged labour which has negative consequences for the newborn [20]. So, while being multiparous is risky to the mother, the infant being born to a primiparous mother is even at a greater risk. l) Page 21: line 395: to **~~their counterparts~~** read: ….This finding is similar to the findings reported in studies conducted in Brazil [17],………. m) Page 21: line 396-397: to read: ……. This could be **~~because~~** attributed to intrauterine growth retardation (IUGR), placental insufficiency, and increased interventional delivery that may be performed regardless of the gestational age. n) Page 21: line 402: to read: ……… than those who were not referred. o) Page 20: line 406: to rea: **~~Based on this~~** It is, therefore, recommended that midwives should prioritize providing timely treatment to those vulnerable newborns. p) Page 21: line 408: to read: The study **~~suggested~~** revealed that……. q) Page 21: line 409: to read: ….. than **~~their peers~~** those mothers who did not have PROM…. r) Page 21: line 410: replace “accordance” with “agreement” s) Age 22: line 416 -417: to read:…….than **~~their counterparts~~** those without fetal malposition. **~~This is a warning to~~** This finding should serve as a guide to midwives to spot **~~it early and consult an obstetrician to take~~** the danger early and call for immediate action to save the lives of the newborns. t) Page 22: line 419: “1.97-fold increased” to read “1.97-fold increase” in the risk of NNM. u) Line 420: delete “**~~than their peers”~~** v) **~~This finding was supported by”~~** to read: This finding is consistent with a study in India which found that highly thick meconium-stained amniotic fluid is a sign of fetal distress [29]. Delete **~~“which is concerning to the neonate’s better prognosis’~~** w) Page 22: line: 426: to read: …… **This is consistent with the findings in our study which showed that neonates born to women who had AFSOL lasting more than 8 hours had a 1.81-fold increased risk of NNM than those born to women who had AFSOL lasting less than 8 hours.** Delete ~~than their counterparts~~ x) Page 22: line 428: to read: “This **is”** **to read** “This may be”….. y) Page 22: line 429: to read: …….. “The most important strategy for **~~intervening~~** early detection of prolonged AFSOL is the consistent use and interpretation of the partograph. z) Page 23: line 439: replace “endorses” with “supported”. Delete: ~~“This is the responsibility of the midwives and obstetricians who work.~~ **It does not make any sense here!.**   **aa**. Page 23 line 442: “lasting” to read “going through”  bb. Page 23: line 448: “indirectly” to read “indirect”  cc. Page 23: line 450: **delete the whole sentence**   dd. **Page 23: line 454: Ethiopian studies supports This finding is supported by other studies conducted in Ethiopia [13, 19]** ee. **Page 24:** line 462: “this idea” to read “this finding” ff. **Page 24: line 466-467: to read:** “The strength of this study lies in the fact that it is a multi-centre study with a large sample size. In addition, a psychometrically validated scale was used to assess the outcome. **The study also determined the direct ……….** gg. **Page 24: line 471-473: …….**were ~~“missed~~” excluded and only live births ~~babies~~ were studied in the early neonatal period with no follow-up. We strongly recommend that a study be conducted to cover the entire neonatal period. hh. **Page 25:** line 478: replace: “nearly dead but living newborn” with “moribund newborn”. ii. **Page 25;** line 479: to read: “educate midwives, doctors and other health professionals on how to manage limited resources **~~in order~~** to improve quality of care and reduce NNM and mortality. | It has been corrected as from line 351 to 356 “The study revealed that the proportion of Neonatal Near-miss was 28.6%. In comparison to studies conducted in other health facilities in Northeastern Brazil [43], Australia [5], and Burkina Faso [3], the proportion was higher.  This could be due to a disparity in the quality of care provided in the health facilities in these countries,.  In addition, prospective follow-up was used in the Brazil and Australia studies, and the outcome was determined using a different measurement scale, whereas in our study, we used a contextualized [38], valid, and reliable NNMAS [39]” in the main document.  “This finding, on the other hand, was consistent with other studies from Brazil (46), and the Jima Zone, southwest Ethiopia [12].” In the man document.  It has been corrected from line359 to 363 in the main document.  “The NNM (rate read as proportion of 28.6% in our study was, however, lower when compared to other studies conducted in health facilities in Brazil [44], central Gujarat India [6], Nepal [7] and Central Uganda [45}, Hawasa Ethiopia [46], south Ethiopia [14], and Debre Tabor hospital, Northwest Ethiopia [13]. Participants in these other studies in Brazil, central Gujarat India, and Uganda, were neonates born to women hospitalized with severe obstetric problems”.  “….and Debre Tabor were conducted on single high-caseload referral hospitals” in line 365.  It has been incorporated as “In contrast, in the studies conducted in Nepal, Hawasa-Ethiopia, and Debretabor Hospital in Northwest Ethiopia” in line 370 of the main document.  “In our study, newborns born to women who could not read and write were…”line 357 in the main document.  “The findings in our study show that the chances of having NNM…” line 379 in the main document.  It has been corrected as from line 381 to 386 in the main document”This is consistent with the findings in a study conducted at Debre Tabor Hospital in Northwest Ethiopia [13], and tends to suggest that psychological concerns, stress, and intense labour pain may have a significant impact on primiparous women's energy and power during labour and birth. This may result in prolonged labour which has negative consequences for the newborn [20]. So, while being multiparous is risky to the mother, the infant being born to a primiparous mother is even at a greater risk”.  It has been corrected as “This finding is similar to the findings reported in studies conducted in Brazil…” line 390 in the main document.  It has been corrected as “This could be attributed to intrauterine growth retardation (IUGR), placental insufficiency, and increased interventional delivery that may be performed regardless of the gestational age” line 391 to 393 in the main document.  It is corrected as” …….to experience NNM than those who were not referred” line 397 in the main document.  “It is, therefore, recommended that midwives should prioritize providing timely treatment to those vulnerable new-borns” in line 401 to 402 in the main document..  “The study revealed that babies born to women who had a premature rupture….” in line 403 in the main document.  “….than those mothers who did not have PROM” line 404 to 405 in the main document..  “The finding is in agreement with those” in line 405 in the main document.  It has been corrected as “likely to develop NNM than those without fetal malposition” line 411 in the main document  “This finding should serve as a guide to midwives to spot the danger early and call for immediate action to save the lives of the new-borns” from line 411 to 413 in the main document.  It has been corrected as” had 1.97-fold increase in the risk of NNM” the main document in line 415.  It has been deleted in the main document.  It has been corrected as “This finding is consistent with a study in India which found that highly thick meconium-stained amniotic fluid is a sign of fetal distress [29]. As a result, early detection and intervention” in line 415 to 417 in the main document.  It has been corrected in the main document as”  This is consistent with the findings in our study which showed that neonates born to women who had AFSOL lasting more than 8 hours had a 1.81-fold increased risk of NNM than those born to women who had AFSOL lasting less than 8 hours” line 421 to 423 in the main document.  “This may be due to the increased likelihood of fetal distress” line 423 to 424 in the main document.  It has been incorporated in the main document as”The most important strategy for early detection of prolonged AFSOL is the consistent use and interpretation of the partograph” line 424 to 426.  “A previous study supported up this claim” in line 434 in the main document.  “….going through an active first stage of labour in less than 8 hours” line 436 in the main document.  “……It has an indirect effect on…”line 442 in the main document  It has been corrected as “This finding is supported by other studies conducted in Ethiopia” line 448 to 449 in the main document  “this finding” corrected in the main document in line 455  It has been corrected in the main document as” The strength of this study lies in the fact that it is a multi-centre study with a large sample size. In addition, a psychometrically validated scale was used to assess the outcome. The study also determined the direct and indirect effects of variables on NNM via mediators” in the main document line 458 to 460.  “….one of the selected hospitals were excluded and only live births were studied in the early neonatal period with no follow-up. We strongly recommend that a study be conducted to cover the entire neonatal period “line 463 to .465 in the main document.  It has been corrected as “…which could indicate a moribund new-born” in the main document line 470 in the main document.  “It can also be used educate midwives, doctors and other health professionals on how to manage limited resources to improve quality of care and reduce NNM and mortality” incorporated in the main document line 470 to 472. |
| 10 | **Conclusion:**   a) **Page 25 line482: “couldn’t” to read “could not”** b) **Page 25: line 488: ….. “could be of supreme importance in reducing NNM.”** | Dear reviewer  Thank you for your significant contribution to the improvement of this paper.   a. It has been corrected as “ could not “ in the main document line 474. b. “Early diagnosis and intervention of these potential danger signs could be of supreme importance in reducing NNM” line 480 in the main document |
| 1 | **Reviewer #3** | Response |
| 1 | Thank you for giving me the opportunity to review this interesting article that affirmed existing knowledge. The authors rigorously statistical analysis of the data is highly commendable. However, there are repetitions of statements that makes the results and discussion pages very long. I suggest reduction of discussion pages by effective narration of results and correlation with other published work. Avoid repeating sentences already detailed in the results pages. A constructive discussion of results and not stating results in the discussion narrations. | Dear reviewer  Thank you very much for your suggestions for improving our work. Based on your and the second reviewer's comments, we addressed the issues, including grammatical correction and the avoidance of the reaptad sentence in all parts, as I mentioned above line by line in each page.  We attempted to condense the result and discussion sections while keeping the main findings intact, despite the fact that the mediation analysis reporting is naturally broad. |
| 2 | The definition of prolonged active phase of labour used in this study may be justified further in relation to parity. | Thank you again for raising this important point. It has been corrected in the main document as “Prolonged active first stage of labor (AFSOL) is defined as the time interval between the latent stage of labor and full cervical dilation that is greater than or equal to 8 hours in both in both parity types. |

**Thank you so much**
